# Critical assessment of the endocrine potential of Linalool and Linalyl acetate: proactive testing strategy assessing estrogenic and androgenic activity of Lavender oil main components

**DOI:** 10.1007/s00204-023-03623-z

**Published:** 2023-10-31

**Authors:** Lars Hareng, Susanne N. Kolle, Caroline Gomes, Steffen Schneider, Markus Wahl

**Affiliations:** BASF SE, 67056 Ludwigshafen, Germany

**Keywords:** Endocrine, Linalool, Linalyl acetate, Lavender, Estrogen, Androgen

## Abstract

The acyclic linear monoterpenes Linalool (Lin) and Linalyl acetate (LinAc) occur in nature as major constituents of various essential oils such as lavender oils. A potential endocrine activity of these compounds was discussed in literature including premature thelarche and prepubertal gynecomastia due to lavender product use. This study aims to follow-up on these critical findings reported by testing Lin and LinAc in several studies in line with current guidance and regulatory framework. No relevant anti-/ER and AR-mediated activity was observed in recombinant yeast cell-based screening tests and guideline reporter gene in vitro assays in mammalian cells. Findings in the screening test suggested an anti-androgenic activity, which could not be confirmed in the respective mammalian cell guideline assay. Mechanistic guideline in vivo studies (Uterotrophic and Hershberger assays) with Lin did not show significant dose related changes in estrogen or androgen sensitive organ weights and a guideline reproductive toxicity screening study did not reveal evident effects on sex steroid hormone sensitive organ weights, associated histopathological findings and altered sperm parameters. Estrous cycling and mating/fertility indices were not affected and no evident Lin-related steroid hormone dependent effects were found in the offspring. Overall, the initial concerns from literature were not confirmed. Findings in the yeast screening test were aberrant from follow-up guideline in vitro and in vivo studies, which underlines the need to apply careful interpretation of single in vitro test results to support a respective line of evidence and to establish a biologically plausible link to an adverse outcome.

**Supplementary Information:**

The online version contains supplementary material available at 10.1007/s00204-023-03623-z.

## Introduction

Linalool (Lin) and Linalyl acetate (LinAc) are both acyclic monoterpenes naturally occurring as constituents of various essential oils (EOs) derived from plants such as lavender, cardamon as well as clary sage and many other (EMEA 1999; Api et al. 2015a, b, 2022). LinAc is the acetic ester of Lin and hydrolyses quickly to release the acyclic linear monoterpene alcohol Lin (OECD SIDS). The respective natural EOs constitute of high amounts of Lin and LinAc and lavender oil is a well-known example containing up to 80% Lin and LinAc depending on the natural impact on the harvest and contributing factors such as region and climate (Wang et al. 2021; Nedeltcheva-Antonova et al. 2022). In addition, the naturally occurring Lin and LinAc can also be produced synthetically. Regardless of their origin, Lin and LinAc are used globally for the application in fragrances for their fresh floral, herbal scent of lavender and other such as rosewood, petitgrain, bergamot. Basically, Lin and LinAc are the “molecular chemical mediators” of the lavender scent olfactory perception (Elsharif et al. 2015).

In addition to their use in fragrances, Lin and LinAc are also flavors which naturally occur in a plethora of plants, spices and fruits. As an example, Lin is the major flavoring agent in tea, citrus fruits, and also in alcoholic beverages. In beer, Lin naturally originates from hop and is responsible for the fruity taste (Api et al. 2015a, b, 2022; Denby et al. 2018) and in grape juice and wine, Lin is a key component among the monoterpenes forming the bouquet varieties (Mateo and Jiménez 2000). With respect to the overall regulatory landscape, Lin and LinAc are both listed as flavours in the EU (EC 1334/2008) and globally (Fukushima et al. 2020). Furthermore, due to their use as fragrance substances in personal care products, Lin and LinAc fall under the Cosmetics Directive within the EU (CPR, EC 1223/2009). Under REACH (EC 1907/2006) legislation, both Lin and LinAc are registered for the manufacture as non-intermediates with a production volume beyond 1000 tons per year in the EU for their fragrance use in home care and air care products.

Available toxicological and ecotoxicological data for Lin and LinAc have been extensively reviewed by RIFM (Api et al. 2015a, b, 2022). In the recent years, a potential endocrine activity of lavender oil and its main components Lin and LinAc has controversially been debated in the literature. Ramsey and coworkers associated the use of lavender oil containing personal care products with premature thelarche and prepubertal gynecomastia in children in the US (Ramsey et al. 2019). The authors presented individual human case reports together with mechanistic investigations as evidence for potentially underlying estrogenic and anti-androgenic “mode(s)-of-action” (MoA) of the observed endocrine activity for Lavender oil as well as Lin and LinAc individually as the main components. Overall, human case studies reported by Ramsey et al. (2019) were discussed as inconclusive due to methodological limitations, possible confounders together with the lack of causality due to not confirmed exposure towards lavender oils and its components Lin and LinAc (Hawkins et al. 2020). Driven by the European “Chemical Strategy for Sustainability” (CSS), endocrine disruption (ED) as a mode-of-action is being implemented as a new and separate hazard class into the CLP (“Classification, Labelling and Packaging” directive, EC 1272/2008). To follow up on the potential mechanistic in vitro studies, that suggested an underlying estrogenic and anti-androgenic mode of action for Lin and LinAc and to add to the dataset required for future classification decisions, we performed an in-depth assessment of available data from the public literature including the EPA Tox21-related EDSP (Endocrine Disruptor Screening Program) database. Furthermore, we conducted a proactive testing strategy guided by EU-requirements, the ECHA and EFSA guidance and the OECD conceptual framework (OECD TG 150), in which Lin and LinAc were tested in estrogen and androgen receptor (ER and AR) related in vitro tests. Confirmatory in vivo tests were performed with the representative hydrolysis product Lin to conclude on the modes of action proposed.

## Materials and methods

### Test materials

For the in vitro and in vivo studies, Linalool (Lin; 3,7-dimethyloctadien-1,6-ol-3; CAS 78-70-6; batch 00179277L0) supplied by BASF SE (Ludwigshafen, Germany) with a confirmed purity of 99.7 g/100 g was used. Linalyl acetate (LinAc; 3,7-dimethyl-1,6-octadien-3-yl-acetate; CAS 115-95-7; batch 00100577L0) supplied by BASF SE (Ludwigshafen, Germany) with a confirmed purity of 99.8 g/100 g was used for the in vitro tests. The control substances 17β-estradiol (CAS 50-28-2), 17α-estradiol (CAS 57-91-0), 17α-ethynylestradiol (CAS 57-63-6), testosterone propionate (CAS 57-85-2), 17α-methyltestosterone (CAS 58-18-4), dihydrotestosterone (CAS 521-18-6), hydroxyflutamide (CAS 52806-53-8), corticosterone (CAS 50-22-6), flutamide (CAS 13311-84-7), bisphenol A (CAS 80-05-7), bis-(2-ethylhexyl)-phthalate (CAS 117-81-7) and cycloheximide (CAS 66-81-9) were purchased from Sigma–Aldrich Chemie GmbH (Schnelldorf, Germany). Tamoxifen (CAS 10540-29-1) was obtained from Sanbio BV (Uden, The Netherlands), (Z)-4-hydroxytamoxifen (CAS 68047-06-3) from Bio connect life sciences (Huissen, The Netherlands), digitonin (CAS 11024-24-1) from Acros organics (Geel, Belgium) and 17α-methylandrostan-17β-ol-3-one (CAS 521-11-9) was ordered from TCI (Zwijndrecht, Belgium). For the Hershberger assay, flutamide was purchased from Spectrum Chemical MfG Corp, New Brunswick NJ, USA. Positive control substance purities were ≥ 98%.

### In vitro human estrogen and androgen receptor activity assays using recombinant yeasts (YES and YAS assays)

Yeast (*Saccharomyces cerevisiae*) stably transformed with the gene coding for hERα and expression plasmids carrying an ERE (YES assay) or with the human androgen receptor (hAR) gene and expression plasmids carrying androgen-responsive sequences (YAS assay) together with the reporter gene *lac*-Z (encoding the enzyme β-galactosidase) was obtained from Professor Vollmer of the Technical University Dresden.

The YES assay was performed as described by Routledge et al. (Routledge and Sumpter 1996) and Kolle et al. (Kolle et al. 2010) with slight deviations. The test chemicals were serially diluted in DMSO in tenfold steps and 2 µL of each concentration was transferred to a 96-well optically flat-bottom microtiter plate, resulting in final test substance concentrations on the plate ranging from 10^–3^ to 10^–9^ mol/L, in the presence or absence of 17β-estradiol (E2) at a concentration of 10^–9^ mol/L (to determine potential ERα antagonistic or agonistic effects of the test substance, respectively). Yeast cells were suspended in cell growth medium and maintained at 32 °C in a shaking incubator (85 rpm). Aliquots (200 µL) of medium containing recombinant yeast and the chromogenic substrate, chlorophenol red-β-D-galactopyranoside (CPRG) were then dispensed to each sample well. Each plate contained at least eight vehicle control (1% DMSO) wells, an E2 standard curve ranging from 10^–6^ to 10^–12^ mol/L, and a positive control for anti-estrogenic (antagonistic) activity, hydroxytamoxifen at a concentration of 10^–6^ mol/L in the presence of 10^–9^ mol/L E2. Each test or control chemical concentration was tested in quadruplicate in at least two independent experiments. Absorbance at 570 nm was measured after 48 ± 4 h incubation using a plate reader. Yeast growth was determined by optical density at 690 nm to monitor cytotoxicity (≤ 50% versus vehicle control). For the evaluation of antagonistic effects only non-toxic test chemical concentrations were taken into consideration. A test chemical was rated to exert agonistic effects when a relative increase in β-galactosidase activity exceeded 15% in at least one concentration in at least two independent runs. Likewise, a test chemical was rated to exert antagonistic effects when a relative decrease of a defined concentration of the positive control in β-galactosidase activity exceeded 20% in at least one concentration. Accordingly, it was considered negative, if these conditions were not met in at least two independent runs.

The YAS assay was performed as described by Sohoni et al. (Sohoni and Sumpter 1998) and Kolle et al. (2010) with slight modifications and essentially as described above for the YES assay with the exception, that concentrations of 5α-dihydroxytestosterone (DHT; 10^–6^ to 10^–11^ mol/L) served as androgen agonistic control and hydroxyflutamide (10^–5^ mol/L) in the presence of DHT (5 × 10^–9^ mol/L) was used as androgen antagonistic control, respectively. The YES and YAS assays were conducted under Good Laboratory Practice—like conditions in a laboratory certified for Good In Vitro Method Practicises.

### In vitro human estrogen and androgen receptor activity assays using mammalian cell lines (ERTA and ARTA according to OECD TG 455 and 458)

The ERTA uses the stably transfected human hERα-HeLa-9903 cells expressing hERα and an hERα inducible luciferase reporter gene. The AR-EcoScreen™ cell line derived from a CHO-K1 cell line stably transfected with hAR expression vector and a firefly luciferase reporter vector bearing four tandem repeats of the androgen responsive element and a stably expressed *Renilla* luciferase reporter construct is used in the ARTA. Both cell lines were obtained from the Japanese Collection of Research Bioresources (JCRB) Cell Bank (Osaka, Japan). ERTA and ARTA experiments were conducted in accordance with the OECD Principles of Good Laboratory Practice following the respective OECD TG 455 and 458 (OECD 2012, 2023).

Cells were grown in cell culture medium and maintained at 37 °C ± 1.0 °C and in a humid atmosphere of 80–100% containing 5% ± 0.5% CO_2_. Cells were seeded in white 96-well plates at a seeding density of 1 × 10^5^ cells/mL in the ER agonist and ARTA assays, and 1.5 × 10^5^ cells/mL were used in the ER antagonist assay. Test and control chemicals were serially diluted in DMSO and further diluted in agonist or antagonist exposure medium (spiked with E2 for ERTA and DHT for ARTA). Cells were exposed for 20 ± 4 h to test chemicals or controls. Lin or LinAc in DMSO (0.1% final concentration) was tested up to a maximum concentration of 10^–3^ M for the ERTA and 10^–4^ M (Lin) or 10^–3.5^ M (LinAc) for the ARTA. To confirm stability of the cellular response, positive controls for agonistic (E2, 17α-estradiol and 17α-methyltestosterone) and antagonistic (tamoxifen) activity, and negative controls for agonistic (corticosterone) and antagonistic (flutamide) activity were applied in a suitable concentration range in ERTA experiments. On each ERTA test plate, positive agonist control (1 nM E2) and vehicle control (VC; 0.1% DMSO) wells were included. On each ER antagonist test plate, positive antagonist (1 µM 4-hydroxytamoxifen), spike-in (25 pM E2) and positive cytotoxicity (100 µM digitonin) control wells were included. Accordingly, concentration ranges for ARTA positive controls for agonistic (DHT and mestanolone) and antagonistic (hydroxyflutamide and bisphenol A) activity and the negative control (di-(2-ethylhexyl)phthalate (DEHP)) were applied. On each ARTA test plate, positive agonist control (10 nM DHT), VC and positive cytotoxicity control wells (10 µg/mL cycloheximide) were included, respectively. On each AR antagonist test plate, positive antagonist (1 µM 4-hydroxytamoxifen) and spike-in (500 pM DHT) control wells were included. Each test or control chemical concentration was tested at least in triplicate in two independent experiments. For detailed test plate layouts see OECD TGs 455 and 458.

The luciferase activity was determined using the standard luciferase assay system in the ER agonist assay and the Steady-Glo Luciferase assay system in the ER antagonist and AR agonist assays (Promega Corporation). In the AR antagonist assay, the Dual-Glo luciferase assay system (Promega Corporation) was used to measure the firefly luciferase and *Renilla* luciferase activity in the same well via a luminometer. All relative light unit values from a plate were normalized to the mean of their respective VC and the relative induction was calculated as the percentage of the maximum induction of the reference positive control (1 nM or 25 pM E2 and 10 nM or 500 pM DHT). For each test and control chemical, the maximum level of response induced by a chemical, expressed as a percentage against the response by the reference positive control on the same plate (RPC_max_) was calculated. If possible, concentrations inducing 10 or 50% of the maximum level of positive control (PC10 or PC50), as well as concentrations inhibiting 30% or 50% of the maximum level of the positive control (IC30 or IC50) were calculated. A test chemical was considered positive in the ER/AR agonist assay, if the RPC_max_ was ≥ 10% of the positive control response and positive in the ER/AR antagonist assay if a log IC30 could be calculated in at least two independent experiments. Cytotoxicity of each test chemical was evaluated in the ERTA using the 3-(4,5-dimethylthiazol-2-yl)-2,5-diphenyltetrazolium bromide (MTT) test under agonist and antagonist conditions. In the ARTA, cytotoxicity was assessed in the antagonist plates by measuring the *Renilla* luciferase activity (constitutively expressed). Treatment groups showing more than 20% cytotoxicity in relation to VCs were considered cytotoxic and excluded from further analysis.

### In vivo mechanistic studies for estrogen and androgen receptor mediated activity (Uterotrophic and Hershberger assay according to OECD TG 440 and 441)

For the Uterotrophic assay (OECD 2007), young adult Crl:CD(SD) female rats (50 days old at receipt) from Charles River Laboratories Inc. (Raleigh, NC, USA) were used 3 days following ovariectomies performed by the supplier. Animals assigned achieved similar group mean body weights and were in persistent diestrus. Two animals were housed together in polyphenylsulfone, solid-bottom cages with heat-treated aspen bedding material (Northeastern Products Corp.), rodent diets with less than 300 ppm genistein equivalent content and suitable environmental enrichment at a temperature of 20–25 °C, 30–70% humidity, and 12-h light/dark cycle. Envigo Laboratories 2016CM Teklad Global 16% Protein Rodent Diet and municipal drinking water were provided ad libitum. Following acclimatization for at least 10 days, 6 animals per group were dosed via oral gavage (100, 300 and 1000 mg Lin/kg bw/d), corn oil as vehicle control or via subcutaneous injection in the scapular region (1 µg 17α-ethynylestradiol/ kg bw/d) as positive control once daily for 3 consecutive days. Mortality, clinical signs, body weight/gains, macroscopic examinations, and uterine weights were determined during the study or after euthanasia by carbon dioxide inhalation approximately 24 h after last dose administration. Each “wet” uterus was weighed intact (with the luminal fluid), then opened longitudinally and blotted with filter paper to remove the luminal fluid and to obtain the blotted uterus weight.

For the Hershberger assay (OECD 2009), peripubertal, castrated male Crl:CD(SD) rats (45 days old at receipt) were received from the same breeder and housed as described above, except for the food provided (PMI Nutrition International, LLC Certified Rodent LabDiet^®^ 5002). Six animals per group were dosed with corn oil as vehicle control, test substance (100, 300 and 1000 mg Lin/kg bw/d) or the anti-androgenic positive control substance (flutamide; 3 mg/kg bw/d) via oral gavage once daily for 10 consecutive days. The androgenic positive control (testosterone propionate; 0.2 mg/kg bw/d) was administered via subcutaneous injection in the dorsal scapular region alone or co-administered with 3 additional Lin dose groups and flutamide. Mortality, clinical signs, body weight/gains, macroscopic findings, and organ weights (adrenal glands, bulbourethral (Cowper’s) glands, glans penis, levator ani and bulbocavernosus (LABC) muscle group, seminal vesicles with coagulating glands, ventral prostate, kidneys, liver) were determined during the study or after isoflurane inhalation and euthanasia by exsanguination approximately 24 h after last dose administration. In both assays, the analyzed dosing formulations were within the protocol-specified range of target concentrations (85 to 115%) for suspensions and were homogeneous.

### In vivo combined repeated dose and reproduction/developmental toxicity screening test in rats according to OECD TG 422

Male and female Wistar rats (Crl:WI(Han); free of signs of disease and nulliparous females on delivery) were supplied by Charles River Laboratories, Research Models and Services, Germany GmbH (Sulzfeld, Germany). The receipt of males (about 77–90 days old) and females (about 70–76 days old) at different age warranted, that no sibling males and females were paired. Animals were housed singly in Makrolon type M III cages, with dust-free wood chip-bedding and suitable environmental enrichment, at a temperature of 20–24 °C, 45–65% humidity, and 12 h light/dark cycle on Kliba mouse/rat maintenance diet with a phytoestrogen content of less than 350 μg genistein equivalents/gram (Provimi Kliba SA, Kaiseraugst, Switzerland) and municipal drinking water ad libitum. All procedures were conducted according to the requirements of Good Laboratory Practice and of DIN EN ISO/IEC 17020 in an AAALAC-approved laboratory in compliance with the animal welfare principles laid down by the German Animal Welfare Act and the effective European Council Directive. OECD test guideline 422 served as basis for the experimental design of the study (OECD 2016). Following an acclimatization period of 21 days (which included 14 days estrous cycle determination in a pool of 50 non-randomized female animals), male and female (all with regular cycles) rats were randomly assigned to groups of 10 animals per sex per dose. F0 and selected F1 animals were administered Lin in corn oil (4 ml/kg body weight) once daily at doses of 50, 200, or 800 mg/kg body weight, or the vehicle alone as control group. Correctness of the applied Lin concentrations was analytically confirmed twice during the study. Treatment commenced at least 14 days before mating, throughout the mating period (males and females), gestation, lactation and post-weaning (females only). Males and females from the same group were paired overnight in a 1:1 ratio for a maximum of 2 weeks. Mating was confirmed by the presence of sperm in a vaginal smear (denoted gestation day (GD) 0) and ceased afterwards. Offspring were sacrificed on postnatal day (PND) 21 or after reaching puberty, while the parental females were sacrificed approximately 14 days post-weaning and the parental males were sacrificed approximately 29 days after the commencement of treatment.

Animals were checked daily for clinically evident signs of toxicity (including gross morphologic findings in the offspring), littering, nesting and lactation behavior of the dams during parturition and lactation. Specific observations included weekly detailed clinical observations, a functional observational battery and motor activity measurement at study end. Food consumption and body weights of the parental and offspring animals were checked regularly.

Hematology and clinical chemistry examinations were conducted in 5 male and 5 female parental animals per group. Sperm quality (motility, sperm head count, morphology) was assessed in all F0 males. Parental animals sacrificed on schedule were subjected to gross pathologic examination. The full organ spectrum required by OECD TG 422 was weighed, fixed in 4% neutral buffered formaldehyde solution or Davidson’s solution and investigated by light microscopy.

Anogenital distance measurements (defined as the distance from the anus [center of the anal opening] to the base of the genital tubercle) were conducted in a blind randomized fashion, using a measuring ocular on all live male and female pups on PND 1. Likewise, all surviving male pups were examined for the presence/absence and number of nipple/areola anlagen on PND 13 and 20.

Serum levels of T4 and TSH were determined in samples from parental males at termination and from pups at PND 13. The concentrations of TSH were determined by radioimmunoassay (RIA), using commercially available RIA test kits and a Gamma-Counter (LB 2111, Berthold, Germany). T4 ELISA was measured with a Sunrise MTP-reader (Tecan AG, Maennedorf, Switzerland) and evaluated with the Magellan-Software of the instrument producer.

Onset of puberty, i.e. day of vaginal opening (females) or balanopreputial separation (males), was recorded (one male & female per litter). All offspring, that survived to scheduled termination including adolescents reaching puberty, and stillborn or decedents before schedule were examined externally, eviscerated, and their organs were examined macroscopically.

## Results

### Identification of estrogen and androgen receptor mediated effects of Linalool (Lin) and Linalyl acetate (LinAc) in vitro

**Fig. 1:**
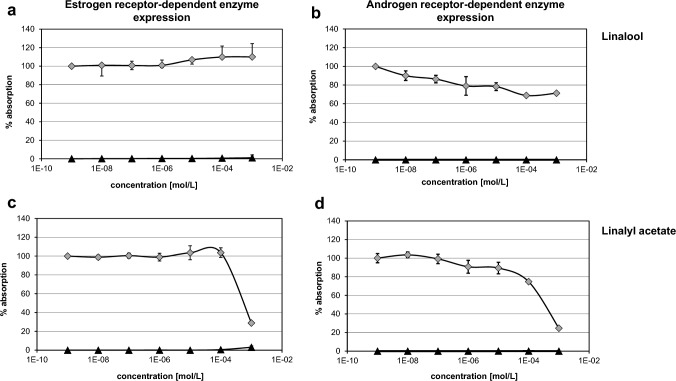
Estrogen and androgen receptor mediated enzyme expression in the YES/YAS assay. ER and AR mediated β-galactosidase activity was measured in the YES assay (**a**/**c**) and YAS assay (**b**/**d**) by detecting colour development at 570 nm after incubation with chlorophenol red-β-D-galactopyranoside (CPRG) for 48 h (± 4 h) together with Linalool (**a**/**b**) or Linalyl acetate (**c**/**d**) at 10^-9^–10^-3^ M in the absence (▲) or presence (◊) of 1 mM estradiol (anti-estrogen related assay) or 5 nM DHT (anti-androgen related assay), respectively. Cell densities were measured at 690 nm to assess turbidity due to yeast growth. Results were expressed as median % absorption compared to the respective positive control activity ± min/max; *n*=4 wells per condition; 2–3 independent runs

Both substances Lin and LinAc were tested in an ED screening test using hER and hAR transfected yeast cells. The expression of estrogen and androgen receptor mediated reporter gene activity was not significantly increased in the presence of Lin or LinAc at concentrations up to 10^–3^ M (see Fig. 1 and Supplementary Table S1). With a maximum mean activity value of 3.1%, the test substance mediated activities were well below the cutoff value for relevant agonistic activity (i.e. 15% of the activity observed for the respective positive controls). Cell viability (percentages of cell density after test substance treatment compared to vehicle controls) was not evidently altered except for the highest LinAc concentration tested in the YES assay. However, the decrease in cell densities was still above the cut-off value representative for relevant cytotoxicity (i.e. 50%). Thus, no biologically relevant ER or AR mediated activity was identified for both test compounds. In the presence of 1 nM estradiol, no relevant reduction of the ER-related activity was observed except for the highest concentration LinAc (10^–3^ M) tested, which also showed a decrease in cell densities (57% compared to the lowest test item concentration plus estradiol) close but still above the cut-off value for cytotoxicity. In the presence of 5 nM DHT, concentration dependent decreases in AR activities were found with absorption values below 80% at ≥ 1 µM for Lin and at ≥ 100 µM for LinAc. Cell densities were not affected in the anti-androgen-related assay at any concentration tested, which indicates an anti-androgenic effect of Lin and LinAc, rather than a general cytotoxic effect under the chosen testing conditions.

**Fig. 2:**
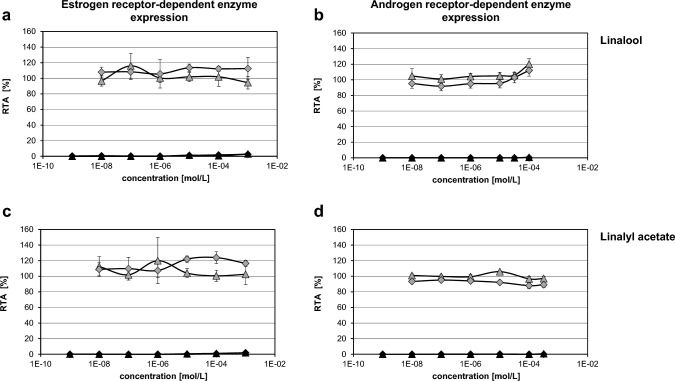
Estrogen and androgen receptor mediated enzyme activity in the ERTA/ARTA assay. Luciferase activity was measured in the ERTA assay (**a**/**c**) and ARTA assay (**b**/**d**) after incubation for 20–24 h with Linalool (**a**/**b**) or Linalyl acetate (**c**/**d**) at 10^–9^–10^–3^ M in the absence (solid) or presence (open grey) of 25 pM E2 (anti-estrogen related assay) or 500 pM DHT (anti-androgen related assay), respectively. The % relative transcriptional activity (RTA) was derived by dividing the normalized relative light units with the mean values of the normalized positive control (1 nM E2; PC = 100%) in the estrogenic assay or with the normalized spike-in control (25 pM E2; spike-in control = 100%) in the anti-estrogenic assay. Accordingly, the mean values of the normalized positive control (10 nM DHT; PC = 100%) or the normalized spike-in control (500 pM DHT; spike-in control = 100%) was used in the androgenic and anti-androgenic assays, respectively. Results are expressed as per cent mean relative transcriptional activity ± SD from two independent experiments (triangles and diamonds, respectively); *n* = 3 wells per condition per experiment

To further clarify the results obtained from the yeast ED screening study, Lin and LinAc were tested in ER and AR-related reporter gene in vitro assays (i.e. ERTA & ARTA) according to OECD TG 455 and 458. As shown in Fig. 2, the incubation of stably transfected hERα-HeLa-9903 with Lin and LinAc at a range from 10^–9^ to 10^–3^ M did not result in a significant and biologically relevant increase in reporter gene activity. The magnitude of the effect (RPC_max_), i.e. the maximum level of response induced compared to 1 nM E2 was 2.6–3.1% for Lin and 1.9% for LinAc and no PC_50_ values could be derived for both test substances (see supplementary Table S2A). The positive control substances E2, 17α-estradiol and 17α-methyltestosterone showed acceptable and consistent responses in the two independent experiments performed, whereas incubations with the negative control substance corticosterone resulted in RPC_max_ (1.0–1.2%) comparable to Lin and LinAc. In the presence of 25 pM E2, a hER mediated activity was not significantly altered by Lin or LinAc up to the highest concentration tested and a log IC30 or log IC50 could not be determined (see supplementary Table S2B). Incubations with the positive control substance tamoxifen resulted in log IC_30_ values of − 6.3 M or log IC_50_ values of approx. − 6.0 M, whereas for the respective negative control flutamide, no such values could be obtained. Overall, these data indicate no direct estrogenic or anti-estrogenic activies of Lin and LinAc under the given testing conditions. Neither Lin nor LinAc were found to be cytotoxic up to the highest concentration tested (see supplementary Table S3A).

Similarly, Chinese hamster ovary (CHO-K1) cells, which were stably transfected with human AR expression vector, did not show any increase in hAR dependent reporter gene activity after incubation with 10^–9^–10^–4^ M Lin or 10^–9^–10^–3.5^ M LinAc. RPC_max_ values ranged between 0.1 and 0.7% for Lin and LinAc and no PC_50_ values could be derived. The negative control substance DEHP resulted in comparable RPC_max_ values of 0.3–1.5% and the positive control substances DHT and mestanolone demonstrated proficiency of the test protocol used. Anti-androgenicity testing by adding 500 pM DHT together with different Lin or LinAc concentrations did not show significant changes and no log IC_30_ or log IC_50_ values could be derived. In line, no such values were obtained for the negative control DEHP and the positive controls HF (log IC_50_ values of approx. − 7.0) and BPA (log IC_50_ values of approx. − 5.5) showed clear anti-androgenic effects. The cytotoxicity in this assay was evaluated by concomitant determination of *Renilla* luciferase activity in the antagonist assay experiments and no cytotoxicity was observed up to the maximum concentrations of Lin and LinAc (see supplementary Table S3B). Thus, Lin and LinAc did not exert direct androgenic or anti-androgenic activies under the conditions of this assay.

### Potential estrogen and androgen receptor mediated activity of Linalool (Lin) in in vivo mechanistic studies

The conflicting findings in the in vitro ED screening tests were further addressed by mechanistic in vivo studies in rats. Lin was chosen as representative substance to be tested further, since LinAc is rapidly hydrolyzed to Lin in vivo. Female ovariectomized rats were treated via oral (gavage) administration of 100, 300 or 1000 mg/kg bw/d in corn oil once daily for 3 consecutive days in an Uterotrophic assay (OECD TG 440). The respective positive control group was treated by subcutaneous injection of 1 µg/kg bw/d 17α-ethynylestradiol (17α-EE) and the negative control group received the vehicle corn oil. Besides mortality, clinical signs, body weights and general macroscopic examinations, the uterine weights were determined to evaluate the ability of Lin to mimic biological estrogenic activities.

All females survived to the scheduled necropsy and clinical observations across all groups were limited to hair loss on the ventral trunk (1 female) in the positive control group. Treatment at the high dose (1000 mg/kg/day) but not at lower doses resulted in reduced mean body weight gains during the dosing period when compared to controls (data not shown). However, the mean absolute body weight was only 3.4% lower than the vehicle control group on the third study day. No internal findings were noted for any female. The treatment with the positive control 17α-EE resulted in lower mean body weight gains or body weight losses and the mean absolute body weight on the third study day was 4.75% lower compared to the vehicle controls. However, no internal findings were noted.

As shown in Table 1, the mean wet and blotted uterine weights were unaffected by Lin administration at any dose. In contrast, significant increases in mean wet (sevenfold versus controls) and blotted (threefold versus controls) uterine weights were observed compared to the vehicle control group after treatment with the positive control 17α-EE. When considering the uterus weights relative to final body weights, no relevant changes were observed after Lin treatment, whereas 17α-EE treatment resulted in increased wet and blotted weights. In conclusion, Lin did not demonstrate biological agonistic estrogenic activities when administered orally to ovariectomized female rats at dose levels up to 1000 mg/kg/day.

**Table 1:** Uterine weights of treated ovariectomized female rats of the Uterotrophic assay (OECD TG 440)

	Negative ctrl	Linalool [mg/kg/d]			positive ctrl
	0	100	300	1000	17α-EE
Uterus—wet					
Mean [g]	0.1055 ± 0.0174	0.1113 ± 0.0190	0.1162 ± 0.0114	0.1109 ± 0.0187	0.7151 ± 0.1255*
Mean [g/100 g bw]	0.036 ± 0.006	0.039 ± 0.007	0.040 ± 0.004	0.039 ± 0.005	0.257 ± 0.047
Uterus—blotted					
Mean [g]	0.0921 ± 0.0120	0.0960 ± 0.0180	0.0999 ± 0.0111	0.0971 ± 0.0158	0.3105 ± 0.0201*
Mean [g/100 g bw]	0.032 ± 0.004	0.034 ± 0.007	0.034 ± 0.003	0.034 ± 0.005	0.112 ± 0.008

Young adult ovariectomized Crl:CD(SD) female rats were dosed via oral gavage with either vehicle (corn oil), 100, 300 or 1000 mg/kg bw/d Linalool once daily for 3 consecutive days. Accordingly, the positive control substance 17α-ethynylestradiol (1 µg/kg bw/d) was administered by subcutaneous injection. Each uterus was dissected and trimmed (retaining the luminal fluid) and “wet” uterus was weighed intact (to the nearest 0.1 mg), then opened longitudinally and blotted with filter paper to remove the luminal fluid to obtain the blotted uterus weight (to the nearest 0.1 mg). Absolute and relative (related to body weight) uterine weights were provided as mean ± SD; *n* = 6 per treatment group

*p ≤ 0.05 (upper tailed *t* test) vs. negative ctrl

The ability of Lin for biological activities consistent with agonism and/or antagonism of androgens in vivo was investigated in a Hershberger assay (OECD TG 441). Male peripubertal and castrated rats were dosed once daily via oral (gavage) for 10 consecutive days with 100, 300 or 1000 mg/kg bw/d Lin in corn oil to assess its androgenic activity. Treatment with the vehicle served as a negative control and 0.2 mg/kg bw/d testosterone propionate (TP) was administered via subcutaneous (bolus) injection as androgenic positive control substance. Anti-androgenic activity was investigated by coadministration of Lin in combination with TP as described above. As an anti-androgenic positive control, flutamide (3 mg/kg bw/d) was administered via oral (gavage) in combination with TP. Next to mortality, clinical signs, body weights and macroscopy, the weights of androgen sensitive organs, i.e. bulbourethral (Cowper’s) glands, glans penis, levator ani and bulbocavernosus (LABC) muscle group, seminal vesicles with coagulating glands and the ventral prostate, were assessed to estimate the anti-/androgenic activity of Lin.

Treatment with Lin and/or the respective control substances was well tolerated, since no test substance-related effects on survival, clinical observations or remarkable macroscopic observations were noted. Increases in mean body weight gains were observed in the TP and (less prominent) in the flutamide/TP positive control group, however no significant substance-related effects on absolute body weights were detectable after treatment in any treatment group (data not shown). Treatment with the high dose of Lin (with and without TP coadministration) resulted in higher liver weights (14.4% and 25.7% relative to the respective control means) whereas no effects on liver, adrenal gland and kidney weights were observed in the other treatment groups including positive controls.

Compared to the vehicle control group, neither statistically significant nor dose dependent differences were observed in androgen dependent organ weights following administration of Lin at dose levels of 100, 300, and 1000 mg/kg/day (Table 2). The androgenic positive control substance (TP) elicited the expected response, resulting in statistically significantly higher mean bulbourethral gland, glans penis, LABC muscle group, seminal vesicle (with coagulating gland) and ventral prostate gland weights compared to the vehicle control group. In addition, the anti-androgenic positive control substance (flutamide) inhibited the androgenic effect of co-administered TP, resulting in statistically significantly lower mean androgen-dependent organ weights compared to the TP positive control group.

**Table 2:** Androgen dependent organ weights of treated orchidoepididymectomized male rats of the Hershberger assay (OECD TG 441)

	Androgenicity				Anti-Androgenicity				
	Negative ctrl	Linalool [mg/kg/d]			Positive ctrl	Linalool [mg/kg/d] + TP			Positive ctrl
	0	100	300	1000	TP	100	300	1000	Flutamide + TP
Bulbo urethral gland	9.5 ± 6.6	9.1 ± 4.6	6.9 ± 2.2	9.2 ± 2.6	27.2 ± 8.3*	32.8 ± 7.7	28.8 ± 4.8	27.6 ± 5.0	9.7 ± 3.0^§^
Glans penis	104.0 ± 14.2	133.7 ± 32.1	117.4 ± 21.7	113.9 ± 34.0	191.6 ± 30.0*	197.6 ± 11.4	190.0 ± 24.4	181.0 ± 11.8	126.7 ± 20.0^§^
Labc muscle GP	166.7 ± 21.2	193.1 ± 52.2	195.5 ± 42.4	196.3 ± 29.9	396.5 ± 84.3*	443.2 ± 75.5	390.7 ± 53.1	402.4 ± 65.9	223.8 ± 43.3^§^
Sem ves/coag Gl	83.4 ± 35.6	115.6 ± 30.1	97.7 ± 16.6	91.0 ± 17.4	380.3 ± 65.4*	423.7 ± 107.7	408.2 ± 90.8	397.5 ± 63.8	122.1 ± 16.0^§^
Ventral prostate	15.4 ± 7.9	19.4 ± 1.7	20 ± 7.9	15.6 ± 2.0	98.8 ± 20.3*	122.8 ± 44.2	116.8 ± 27.2	114.2 ± 42.5	20.9 ± 10.1^§^

Peripubertal, orchidoepididymectomized male Crl:CD(SD) rats were dosed via oral gavage with either vehicle (corn oil), 100, 300 or 1000 mg/kg bw/d Linalool once daily for 10 consecutive days in the absence (androgenic agonism protocol) or presence (androgenic antagonism protocol) of subcutaneously injected testosterone propionate (200 µg/ kg bw/d). Accordingly, testosterone propionate treatment alone served as androgenic positive control substance and administration of flutamide (3 mg/kg bw/d) via oral gavage in the presence of testosterone propionate served as anti-androgenic positive control. Absolute organ weights were provided as mean ± SD; *n* = 6 per treatment group

*LABC MUSCLE GP* levator ani and bulbocavernosus muscle group, *SEM VES/COAG GL* seminal vesicles with coagulating glands

*p ≤ 0.05 (*t* test) vs. negative ctrl

^§^*p* ≤ 0.05 (*t* test) vs. positve (TP) ctrl

Thus, no evidence of androgen agonism or antagonism was noted in peripubertal, castrated male Crl:CD(SD) rats following oral administration of Lin up to the limit dose of 1000 mg/kg/day.

### Potential estrogen and androgen receptor mediated activity of Linalool (Lin) in an in vivo combined repeated dose and reproduction/developmental toxicity screening test in rats

Along with testing of the integrity and performance of the reproductive systems, endocrine-related activity of Lin was assessed in a combined repeated dose and reproduction/developmental toxicity screening test in rats according to OECD TG 422. Estrogen and androgen receptor sensitive endpoints investigated are listed in Table 3.

**Table 3:** Estrogen and androgen dependent endpoints of male (A) and female (B) rats treated in a combined repeated dose and reproduction/developmental toxicity screening test (OECD TG 422)

A										
Males	Endpoint		Negative vehicle control		Linalool [mg/kg/d]					
					50		200		800	
			Mean	± SD	Mean	± SD	Mean	± SD	Mean	± SD
Terminal body weight (F0)		g	419.49	17.27	418.28	29.84	408.58	25.06	381.96^**§§**^	20.19
Organ weights (F0)	Testes	Absolute (g)	3.755	0.332	3.755	0.167	3.679	0.325	3.756	0.140
		Relative (%)	0.896	0.074	0.901	0.065	0.900	0.058	0.985**	0.049
	Epididymides	Absolute (g)	1.223	0.093	1.201	0.104	1.173	0.111	1.186	0.067
		Relative (%)	0.292	0.021	0.288	0.029	0.287	0.024	0.311	0.019
	Seminal vesicle	Absolute (g)	1.594	0.257	1.542	0.152	1.516	0.209	1.366	0.240
		Relative (%)	0.380	0.054	0.37	0.044	0.372	0.051	0.357	0.055
	Prostate	Absolute (g)	1.138	0.161	1.134	0.147	1.114	0.128	0.946**	0.107
		Relative (%)	0.271	0.039	0.272	0.034	0.274	0.037	0.247	0.023
Sperm parameters (F0)	Motile sperm	%	85	5	86	5	86	3	86	6
	Spermatid head count (testis)	Mio/g	115	19	n.d	–	n.d	–	119	24
	Sperm head count (cauda epididymis)	Mio/g	624	192	n.d	–	n.d	–	621	190
	Abnormal sperm	%	5	0.0	n.d	–	n.d	–	5.1	0.3
Anogenital distance	PND 1 males (F1)	mm	3.26	0.17	3.21	0.12	3.04	0.19	3.36	0.45
Anogenital Index		Cubic root	1.69	0.07	1.69	0.08	1.60	0.10	1.84^**§**^	0.20
Nipples (F1)	PND 13 males	Number	1.92	1.00	2.33	0.81	2.39	0.93	2.98	1.66
	PND 20 males	Number	0	0	0	0	0	0	0	0
Preputial separation (F1)	PND	Day	41.1	1.7	40.7	1.3	41.4	1.3	43.2	4.9
	Body weight	g	176.8	9.2	175.5	8.3	178.5	11.3	171.3	8.6

B										
Females	Endpoint		Negative vehicle control		Linalool [mg/kg/d]					
					50		200		800	
			mean	± SD	mean	± SD	mean	± SD	mean	± SD
Terminal body weight (F0)		g	238.57	9.60	237.40	12.67	238.13	7.85	237.57	10.16
Organ weights (F0)	Ovary	Absolute (mg)	119.6	19.8	122.1	18.8	122.8	21.7	125.3	13.3
		Relative (%)	0.050	0.008	0.051	0.007	0.052	0.009	0.053	0.006
	Uterus	Absolute (g)	0.904	0.269	0.757	0.338	0.856	0.268	0.611*	0.098
		Relative (%)	0.379	0.112	0.319	0.145	0.360	0.112	0.258	0.046
Estrous cycling (F0)	Nr. of cycles		2.8	0.4	2.9	0.3	2.9	0.3	2.9	0.3
	Cycles length	Days	3.9	0.2	3.8	0.2	3.8	0.2	3.9	0.2
Anogenital distance	PND 1 females (F1)	mm	1.54	0.08	1.51	0.05	1.49	0.07	1.64	0.23
Anogenital index		Cubic root	0.81	0.04	0.81	0.03	0.80	0.04	0.91^§§^	0.11
Vaginal opening (F1)	PND	Day	29.2	1.8	30.4	1.6	31.6	3.1	28.9	0.6
	Body weight	g	91.4	7.1	96.8	6.4	100.8	15.5	80.6	9.0

Lin was administered daily as a preparation in corn oil to Wistar rats by gavage at doses of 50, 200 and 800 mg/kg bw/d and to selected F1 rearing animals. Control animals were dosed daily with corn oil. The duration of treatment covered a 28 days in-life period in the males and a 2 weeks premating, mating, gestation and lactation period, until approximately 2 weeks after weaning in F0 females. F1 offspring was dosed until sexual maturity was reached. Results were provided as mean ± SD; *n* = 10 parental (F0) animals per sex and treatment group

*n.d.* not determined

*p ≤ 0.05

**p ≤ 0.01 (Kruskal–Wallis test and Wilcoxon test) vs. control group

^§^*p* ≤ 0.05

^§§^*p* ≤ 0.01 (Dunnett test) vs. control group

Absolute mean male reproductive organ weights (testis, epididymides, seminal vesicles) were not affected by treatment up to the high dose of 800 mg/kg bw/day except for a significant decrease in mean prostate weights (− 17% vs. controls). However, the mean terminal body weights were also decreased in this dose group, prostate weights relative to body weights were not significantly decreased and the absolute weights obtained were found to be in the historical control range (0.914–1.274 g). A significant increase in mean relative testes weights (+ 10% vs controls) was observed in high dose animals but still within the historical control range (0.84–0.99% relative to body weight). Furthermore, no Lin-related adverse histopathological changes were observed for prostate and testes and stages of spermatogenesis were comparable to those of the controls. Sperm parameters obtained in spermanalysis, such as sperm head counts in the testis and cauda epididymidis as well as sperm motility and the incidence of abnormal sperms, was not influenced by Lin treatment. Concerning male offspring, the anogenital index (AGD in relation to cubic root of pup weight) was significantly increased in the high dose group (also observed for female offspring), whereas the anogenital distance was unaffected by Lin treatment, indicative for a secondary finding due to lower pup weights. Sex distribution and sex ratios of live F1 pups on the day of birth and PND 21 did not show substantial differences between the control and treated groups (data not shown). The number and percentage of male pups having areolae was not influenced by Lin when examined on PND 13 and during re-examination on PND 20. Male sexual maturation (preputial separation) occurred between PND 38 and PND 55 and the mean number of days as well as the respective body weights did not significantly differ between dose groups and controls.

Concerning female reproductive organs, absolute mean uterus weights were significantly decreased in high dose females (− 32% vs. controls) but still within the historical control range (0.527–0.880 g), while the mean absolute weight in the control group was above that range. Furthermore, the relative mean uterus weights were only slightly but not statistically significantly decreased, and histopathology did not reveal any adverse uterus effects. Ovary weights were not affected by Lin treatment and different stages of functional bodies in the ovaries were present and comparable to the control animals. Estrous cycle data, generated during the last 2 weeks prior to mating for the F1 litter, revealed regular cycles and the mean estrous cycle durations were similar. Sexual maturation (i.e. vaginal opening) occurred between PND 27 and PND 37. Although the body weight of the high -dose female adolescents at puberty was below the concurrent control value, neither a statistically significant nor a toxicologically relevant effect was noted for this endpoint.

Male and female mating indices were not affected, respective fertility indices ranged between 90 and 100% without showing any relation to dosing and no substance-related effects in the gestation index and gestation duration was evident. Further substance-related findings with no direct relation to estrogen and androgen receptor mediated activity such as thyroid-related effects, general systemic and reproductive/developmental toxicity were observed, but presented only briefly (see supplementary Table S4A–C).

The high dose animals showed clear clinical signs and impairment of well-being during various study phases. Decreases in food consumption were observed and body weights in males were below the concurrent control during treatment, whereas in females, they were below control values for most part of the lactation.

Significant changes in serum calcium, sodium, urea, total protein, albumin and cholesterol levels in high dose animals were indicative for an altered liver cell metabolism. In line, liver weights were increased but considered non-adverse due to the absence of a histopathological correlate. The kidneys represented a male specific target organ, showing significantly increased relative weights (high dose only) together with histopathological changes (accumulation of eosinophilic droplets and basophilic tubules, casts) in all dose groups. However, these findings were assumed as non-adverse since they were also present in control animals.

Concerning thyroid-related parameters assessed, a significant drop in mean T4 values was found in parental males. This was not considered a Lin treatment related finding since it did not occur in offspring males and females on PND13 and the respective TSH levels were not significantly affected in both generations. Furthermore, the parents did not show changes in thyroid weights and histopathology findings were comparable to control findings in the parent animals.

Concerning offspring development, the implantation was not affected and indicators of intrauterine embryo-/feto-lethality obtained for all Lin treated dose groups did not show a significant test substance-induced effect compared to controls. Furthermore, the mean number of F1 pups delivered per dam remained unaffected. However, perinatal mortality was indicated in the high dose group by a relevant lower live birth index, an increased number of stillborn pups and a higher number of litters with stillborn pups (see supplementary Table S4B). A reduced viability index (PND 0-4) was observed due to a higher number of dead/cannibalized pups in this dose group, whereas the lactation index (pup survival PND 4-21) was not affected in any dose group. The pup body weight development was also affected in the high dose group during lactation, as it was statistically significantly below the concurrent control throughout the lactation period.

## Discussion

Several case reports of prepubertal gynecomastia and or premature thelarche in children were reported in literature to conincide with a history of continuous exposure to lavender-containing fragrances (Henley et al. 2007; Linklater and Hewitt 2015; Diaz et al. 2016; Ramsey et al. 2019). Lavender oil, along with its main components Lin and LinAc were tested in transfected MCF7 cells (ER) and MDA-kb2 cells (AR) and the authors reported a weak estrogenic effect (starting at 100 µM Lin and 500 µM LinAc) and anti-androgenic effect (starting at 1 µM Lin and 100 µM LinAc) according to these findings. To further elucidate and to complete the lines of evidence for the postulated endocrine activity of Lin and LinAc in terms of E- and A-modality, several in vitro and in vivo studies were conducted in line with the current guidance and the applicable regulatory framework (Andersson et al. 2018; OECD 2018). Accordingly, the aim of the present study was to clarify the lines of evidence for a related adverse effect and establish the respective biologically plausible link for the postulated endocrine activity and the adverse outcome.

The increase in ER activation in vitro for both substances could not be confirmed in our ED screening test using transfected yeast cells (YES) or the guideline ER-related reporter gene (ERTA) assay when tested up to the effective concentrations. A decrease in estradiol mediated reporter activity in the YES assay (only observed for LinAc at 1000 µM) was not confirmed in the ERTA assay performed or reported in literature. This potentially anti-estrogenic effect in the YES assay was seen at a concentration affecting cell densities and the test system is not validated at test concentrations above 100 µM. Therefore, assay intereference is indicated for LinAc incubations at this high concentration. Further in vitro data relating to E-modality in literature were screened and compared to the dataset obtained. Lin did not show anti-/estrogenic activity when tested in the YES assay up to 3.4 mM (Howes et al. 2010). Considering the data generated by the EPA ToxCast program and the AUC scores of the respective computational network model for true agonist and antagonist activity, Lin resulted in agonistic and antagonistic AUC values of 0 due to the absence of any activity in any ER-related in vitro assay (Judson et al. 2015). A few ER binding assays (NVS_NR_bER and NVS_NR_hER) were found active when incubated with LinAc, however, the related assays further downstream of the ER pathway were inactive and the computational network model indicated an interference on the level of the radiolabeled ligand binding assays. Thus, the ER agonistic and antagonistic AUC values were 0 and 0.0001, respectively, and demonstrated an absent or very low likelyhood of a true ER agonist/antagonist activity.

A putative anti-androgenic activity reported in literature was observed in our ED screening test in yeast (YAS) at comparable effect concentrations, however, no such activity was observed in the guideline AR-related reporter gene (ARTA) assay up to test concentrations significantly above the effect concentrations reported. When taking into account the data of the EPA ToxCast program for AR-related activity and the respective computational network model, no relevant antagonistic AR activity was reported for Lin and LinAc and the antagonistic AUC values were 0, respectively (Judson et al. 2020). Accordingly, a true agonistic AR activity is highly unlikely based on the the AUC values of 0 for Lin and 0.068 for LinAc. Associated in silico approaches (CERAPP and COMPARA) identified the structures of Lin and LinAc as inactive against the androgen or estrogen receptor. Concerning the steroidogenesis pathway, Lin and LinAc were also inactive in the aromatase assay according to the EPA database.

Overall, the outcome from the different E- and A-modality related in vitro assays (found in literature and reported in this study) are not fully consistent particularly regarding anti-androgenicity. However, the regulatorily accepted guideline in vitro tests and the integrative network models do not confirm the initial concern of estrogenic and anti-androgenic activity of Lin and LinAc, that was made responsible for the prepubertal gynecomastia or premature thelarche cases published. In line with the OECD conceptual framework for ED testing, Level 3 follow-up in vivo studies were performed and did not confirm any androgen agonism or antagonism or estrogenic activity of Lin. To substantiate the set of mechanistic information and to elucidate potential adverse effects on endocrine relevant endpoints (i.e. Level 4 of the OECD Conceptual Framework), Lin was tested in an combined repeated dose and reproduction/developmental toxicity screening test, in which the offspring rats were treated and investigated until sexual maturity. Sex steroid hormone sensitive parameters—such as count of nipple/areola anlagen or the timing of puberty (vaginal opening and preputial separation)—were not significantly influenced by Lin treatment and most F0 parental animals across all test groups proved to be fertile. Furthermore, no impact on estrus cyclicity, sperm parameters, mating behavior, conception, implantation, intrauterine survival and parturition was observed. While sex steroid hormone sensitive reproductive organ weights (i.e. epididymides, seminal vesicles, ovaries) were not altered, decreases in prostate, testes, uterus weights were found but remained within historical control ranges. In addition, associated histopathological examinations did not reveal Lin related effects indicative for E-, A-, or S-modality related adversity.

When comparing the apparently inconsistent Lin and LinAc in vitro data and the absence of the postulated E/-A-mediated endocrine activity in vivo, it is strongly recommended to apply careful interpretation of single in vitro test results to support a respective line of evidence and to establish a biologically plausible link to an adverse outcome. Cytotoxicity also plays an important role in the interpretation of in vitro test results, which is well accepted for many other endpoints. Thus, it should be well documented in general and taken carefully into consideration for the whole “weight-of-evidence” when assessing in vitro data.

Concerning the reported cases of prepubertal gynecomastia and or premature thelarche, the lines of evidence for E- or A-related endocrine activity, the resulting adversity and the biologically plausible link between those two cannot be established for the lavender components Lin and LinAc.

Furthermore, the proposed link between lavender essential oil exposure and endocrine disruption in children based on the cited case studies is questionable due to poor methodological reliability for participant inclusion and reporting, the presence of other potential ED chemicals in the products used and the multifacturality or different alternative etiologies for the endpoints premature thelarche and gynecomastia (e.g. drugs, diet or environmental exposures). A review of the case reports confirmed little to no epidemiological evidence, that lavender essential oil is capable of causing prepubertal gynecomastia or premature thelarche in children (Hawkins et al. 2020).

Dermal (dorsal) exposure with lavender oil was found non-active in a percutaneous Uterotrophic bioassay in immature female rats, thus proposing no evidence of any estrogenic activity (Politano et al. 2013). It was discussed, whether a sufficient absorption of lavender oil constituents took place in this Uterotrophic assay to produce a distant tissue estrogen effect and that the exposure in the children affected was suspected in the breast area (i.e. near the target tissues) over longer periods (Ramsey et al. 2019). Our in vivo studies were performed via repeated oral bolus exposures partly over several weeks at maximum doses up to 1000 mg Lin/kg bw/d, which represents an evident systemic worst-case exposure via the gastrointestinal tract, compared to Lin in lavender oil fragranced cosmetic products applied on the skin as natural barrier. Although no systemic uptake was monitored in our studies, literature data show an extensive oral absorption after gavage administration, i.e. min 85% of Lin dosed based on excreted amounts in urine and expired air after 72 h (Parke et al. 1974). When taking into account the evident bioavailability observed in the oral gavage studies, we consider it very unlikely, that the absence of adverse effects on E and A sensitive organs in our studies was due to a lack of systemic exposure.

The cosmetic products used in the human case studies contain a broad variety of ingredients including lavender oil, which by itself is a complex mixture of various plant secondary metabolites. Lower transcriptional activation was described when using composed mixtures vs natural lavender oil in the in vitro assays, indicative for other putative components with estrogenic activity. There was a further debate, whether other components than Lin and LinAc or even lavender oil related fragrance compounds were causative for the in vitro findings and the prepubertal gynecomastia or premature thelarche observed in the human cases (Giroux and Orjubin 2020; Tyler Ramsey et al. 2020; Larkman 2020; Ramsey et al. 2020). Our data do not confirm the lavender oil components Lin and LinAc as components of any relevance for the postulated cause and other factors seem to be responsible for the clinical situations described in the case reports.

Although a detailed discussion on thyroid-modality related data is beyond the scope of this publication, such data were obtained in the wake of our testing to clarify the E- and A-modality related concerns. According to our screening reproductive toxicity study, no consistent pattern in thyroid related hormone changes and no adversity in terms of thyroid related histopathology was observed, which does not indicate a T-modality related adverse effect of Lin.

Furthermore, the Level 4 reproductive screening study assessed additional sensitive—but not diagnostic of E-, A- and S-modality—related parameters. Exclusively developmental parameters (i.e. perinatal mortality, reduced early pup survival and pup body weight development) were found to be affected by high dose Lin treatment. This was only observed in the high dose Lin treated animals, which showed a combination of systemic toxicity findings (clinical observations, reduced food consumption and body weights, clinical chemistry changes) indicative of a considerable distress in the parent and offspring rat generations. The foetal and pup survival findings were diffuse and clustered in single litters. Although a relationship of these perinatal offspring losses to treatment seems likely and losses appear as secondary to maternal toxicity, the low number of litters examined in this screening study make a final conclusion difficult. Thus, follow-up testing of Lin or LinAc is considered mandatory to finally conclude on the relevance of the effects observed.

As a resume, we did not detect a consistent estrogen or androgen receptor-related activity of Lin and LinAc in vitro and no E- and A-related adversity of Lin in vivo, as suggested by case reports on lavender products and in vitro assays in literature. Furthermore, our findings in the yeast screening test were not confirmed by our follow-up guideline in vitro and in vivo study results. Thus, we conclude, that the assessment of these modalities should not solely rely on single mechanistic in vitro test data but should instead build on regulatorily acceped in vitro or, if necessary, in vivo studies to inform a respective line of evidence and to establish a biologically plausible link to a well characterized adverse outcome.

### Supplementary Information

Below is the link to the electronic supplementary material.

Supplementary file1 (DOCX 62 KB)

## Data Availability

The authors declare that the data supporting the findings of this study are available within the paper and its Supplementary Information files.
