# Supplementary material for: Critical assessment of the endocrine potential of Linalool and Linalyl acetate: proactive testing strategy assessing estrogenic and androgenic activity of Lavender oil main components
Source: Arch Toxicol. 2023 Oct 31;98(1):347–61. doi: 10.1007/s00204-023-03623-z (PMC10761525; doi:10.1007/s00204-023-03623-z)
Supplement: Supplementary file 1 — Supplementary file1 (DOCX 62 KB) [file 204_2023_3623_MOESM1_ESM.docx]

**Supplementary Information (SI)**

**Table S1A. Overview of agonistic and antagonistic effects induced by Linalool in the YES & YAS assay.**

|  | **YES** | | | | **YAS** | | | |
| --- | --- | --- | --- | --- | --- | --- | --- | --- |
|  | **Agonistic  assay** | **Antagonistic  assay** | **Cell density** | | **Agonistic  assay** | **Antagonistic  assay** | **Cell density** | |
| **Concentration** | **Lin** | **Lin  + Estradiol (1nM)** | **Lin** | **Lin  + Estradiol (1nM)** | **Lin** | **Lin  + DHT (5 nM)** | **Lin** | **Lin + DHT (5 nM)** |
| **[mol/l]** | **[%]** | **[%]** | **[%]** | **[%]** | **[%]** | **[%]** | **[%]** | **[%]** |
| **1.00E-09** | 0.1 | 0.0 | 102.4 | 112.7 | 0.0 | 0.0 | 99.2 | 74.0 |
| **1.00E-08** | 0.2 | 0.9 | 103.4 | 111.1 | 0.0 | -10.0 | 103.0 | 87.0 |
| **1.00E-07** | 0.2 | 0.7 | 104.3 | 114.4 | 0.0 | -13.6 | 102.3 | 82.3 |
| **1.00E-06** | 0.4 | 0.9 | 107.9 | 111.3 | 0.0 | **-21.0** | 99.7 | 87.8 |
| **1.00E-05** | 0.4 | 6.8 | 109.5 | 100.0 | 0.0 | **-21.7** | 110.3 | 89.3 |
| **1.00E-04** | 0.7 | 9.9 | 117.6 | 105.5 | 0.0 | **-31.1** | 112.0 | 96.4 |
| **1.00E-03** | 1.1 | 10.0 | 121.0 | 100.6 | 0.0 | **-28.7** | 107.7 | 92.0 |

**Table S1B. Overview of agonistic and antagonistic effects induced by Linalylacetat in the YES & YAS assay.**

|  | **YES** | | | | **YAS** | | | |
| --- | --- | --- | --- | --- | --- | --- | --- | --- |
|  | **Agonistic  assay** | **Antagonistic  assay** | **Cell density** | | **Agonistic  assay** | **Antagonistic  assay** | **Cell density** | |
| **Concentration** | **LinAc** | **LinAc  + Estradiol (1nM)** | **LinAc** | **LinAc  + Estradiol (1nM)** | **LinAc** | **LinAc  + DHT (5 nM)** | **LinAc** | **LinAc  + DHT (5 nM)** |
| **[mol/l]** | **[%]** | **[%]** | **[%]** | **[%]** | **[%]** | **[%]** | **[%]** | **[%]** |
| **1.00E-09** | 0.0 | 0.0 | 102.9 | 97.0 | 0.0 | 0.0 | 105.7 | 79.5 |
| **1.00E-08** | 0.0 | -1.2 | 100.1 | 92.5 | 0.0 | 3.5 | 106.1 | 83.2 |
| **1.00E-07** | 0.0 | 0.4 | 98.5 | 92.7 | 0.0 | -0.8 | 103.1 | 86.3 |
| **1.00E-06** | 0.0 | -1.1 | 103.1 | 99.3 | 0.0 | -9.3 | 107.0 | 91.9 |
| **1.00E-05** | 0.0 | 3.5 | 99.2 | 98.4 | 0.0 | -10.6 | 107.4 | 91.2 |
| **1.00E-04** | 0.5 | 3.7 | 102.9 | 101.7 | 0.1 | **-25.3** | 109.4 | 98.8 |
| **1.00E-03** | 3.1 | -71.1 | 67.0 | 56.7 | 0.0 | **-75.3** | 102.7 | 91.5 |

**Table S2A. Results of positive / negative controls and test substances in the ERTA/ARTA (estrogen/androgen agonistic assays).**

RPC_max_ = maximum level of response induced by the compound, expressed as percentage of the response induced by the positive control (1 nM E2 in ERTA or 10 nM DHT in ARTA).

PC_50_ = concentration inducing 50% of the maximum level of the positive control (1 nM E2 in ERTA or 10 nM DHT in ARTA)

| **Test type** | **Control/Test Item** | **Experiment** | **Log PC_50_ [M]** | **RPC_max_** |  |
| --- | --- | --- | --- | --- | --- |
|  |  |  |  |  |  |
| ERTA (agonistic assay) | E2 | 1 | -10.8 | 100.0% |  |
|  |  | 2 | -11 | 103.0% |  |
|  | 17α-Estradiol | 1 | -8.7 | 91.5% |  |
|  |  | 2 | -9 | 103.0% |  |
|  | 17α-methyltestosterone | 1 | -5.3 | 57.2% |  |
|  |  | 2 | -5.2 | 54.6% |  |
|  | Corticosterone | 1 | - | 1.2% |  |
|  |  | 2 | - | 1.0% |  |
|  | Linalool | 1 | - | 3.1% |  |
|  |  | 2 | - | 2.6% |  |
|  | Linalyl acetate | 1 | - | 1.9% |  |
|  |  | 2 | - | 1.9% |  |
| ARTA (agonistic assay) | DHT | 1 | -11.01 | 96.9 |  |
|  |  | 2 | -10.04 | 99.4 |  |
|  | Mestanolone | 1 | -9.83 | 87 |  |
|  |  | 2 | -9.97 | 90.9 |  |
|  | DEHP | 1 | - | 0.3 |  |
|  |  | 2 | - | 1.5 |  |
|  | Linalool | 1 | - | 0.7 |  |
|  |  | 2 | - | 0.1 |  |
|  | Linalylacetat | 1 | - | 0.7 |  |
|  |  | 2 | - | 0.2 |  |

**Table S2B. Results of positive / negative controls and test substances in the ERTA/ARTA (estrogen/androgen antagonistic assays).**

Log IC_30/50_ = concentrations of 30%/50% inhibition of transcriptional activity induced by 25 pM E2 (ERTA) or 500 pM DHT (ARTA), respectively.

| **Test type** | **Control/Test Item** | **Experiment** | **Log IC_30_ [M]** | **Log IC_50_ [M]** |
| --- | --- | --- | --- | --- |
| ERTA (antagonistic assay) | Tamoxifen | 1 | -6.35 | -5.97 |
|  |  | 2 | -6.33 | -6.02 |
|  | Flutamide | 1 | - | - |
|  |  | 2 | - | - |
|  | Linalool | 1 | - | - |
|  |  | 2 | - | - |
|  | Linalyl acetate | 1 | - | - |
|  |  | 2 | - | - |
| ARTA (antagonistic assay) | HF | 1 | -7.61 | -7.14 |
|  |  | 2 | -7.31 | -6.88 |
|  | BPA | 1 | -5.92 | -5.6 |
|  |  | 2 | -5.81 | -5.51 |
|  | DEHP | 1 | - | - |
|  |  | 2 | - | - |
|  | Linalool | 1 | - | - |
|  |  | 2 | - | - |
|  | Linalyl acetate | 1 | - | - |
|  |  | 2 | - | - |

**Table S3A. Cytotoxicity data of the ERTA (estrogen agonistic and antagonistic assay).**

VC: vehicle control (0.1% DMSO), PC_MTT_: positive control for cytotoxicity, n.d.: not determined

|  |  | Agonistic assay | | | | Antagonistic assay | | | |
| --- | --- | --- | --- | --- | --- | --- | --- | --- | --- |
|  |  | Cell viability (%) | | | | Cell viability (%) | | | |
| Sample | Concentration | Experiment 1 | | Experiment 2 | | Experiment 1 | | Experiment 2 | |
|  |  | Average | SD | Average | SD | Average | SD | Average | SD |
| VC | 0.1 % DMSO | 100 | 2.6 | 100 | 8.2 | 100 | 15.6 | 100 | 1.5 |
| PC_MTT_ | VC + MeOH | 0 | 0.3 | 0 | 0.2 | 0 | 0.1 | 0 | 0.1 |
| Linalool | 1 mM | 96.3 | 5.7 | 101.9 | 15.2 | 103 | 7.2 | 110.8 | 9.6 |
|  | 100 µM | 99.6 | 3.3 | 102.3 | 13.9 | 101.3 | 2 | 119.7 | 2.5 |
|  | 10 µM | 100.5 | 5.7 | 99.2 | 12.8 | 101.6 | 4.7 | 111.7 | 5.1 |
|  | 1 µM | 101.9 | 1.9 | 105.8 | 25.5 | 107.2 | 14.3 | 112.2 | 4.6 |
|  | 100 nM | 103.8 | 2.5 | 99.6 | 9.2 | 102.3 | 12.1 | 110.9 | 3 |
|  | 10 nM | 100.9 | 6.3 | 112.1 | 6.1 | 98.9 | 0.8 | 112.3 | 7 |
|  | 1 nM | 104.7 | 12.5 | 102.3 | 4.7 | n.d. | n.d. | n.d. | n.d. |
| Linalyl acetate | 1 mM | 100.8 | 10 | 101.7 | 2.8 | 109.9 | 11 | 115.8 | 1.8 |
|  | 100 µM | 108.7 | 7.1 | 98.1 | 7.3 | 111 | 9.7 | 112.7 | 13.1 |
|  | 10 µM | 104.3 | 2.5 | 105.2 | 12.4 | 100 | 21 | 110.6 | 12.8 |
|  | 1 µM | 99.7 | 7.6 | 103.7 | 12.4 | 104.2 | 12 | 113.2 | 14.3 |
|  | 100 nM | 110.7 | 14.5 | 98.6 | 15.7 | 105 | 18.8 | 110.3 | 11.9 |
|  | 10 nM | 101.8 | 1.4 | 102.2 | 23.8 | 106.6 | 8.9 | 106.8 | 7.8 |
|  | 1 nM | 98 | 5 | 106.1 | 6.2 | n.d. | n.d. | n.d. | n.d. |

**Table S3B. Cytotoxicity data of the ARTA (androgen antagonistic assay).**

VC: vehicle control (0.1% DMSO), PC_CT_: positive control for cytotoxicity

|  |  | Cell viability (%) | | | |
| --- | --- | --- | --- | --- | --- |
| Sample | Log Conc. [M] | Experiment 1 | | Experiment 2 | |
|  |  | Mean | SD | Mean | SD |
| VC | 0.1 % DMSO | 108 | 4.8 | 104.2 | 4.8 |
| PC_CT_ | Cycloheximide [10 µg/mL] | 0 | 0.9 | 0 | 1.2 |
| Linalool | -4 | 93 | 5.3 | 93.5 | 3.9 |
|  | -4.5 | 107.8 | 6.7 | 101.8 | 2.1 |
|  | -5 | 97.3 | 3.5 | 100.4 | 7 |
|  | -6 | 114.1 | 4.7 | 102.6 | 7.5 |
|  | -7 | 106 | 4.7 | 102.4 | 6.1 |
|  | -8 | 92.8 | 14.4 | 103.1 | 6.7 |
| Linalyl acetate | -3.5 | 96.9 | 4.8 | 107.7 | 16.9 |
|  | -4 | 114.8 | 2.3 | 107.3 | 7.1 |
|  | -5 | 101.7 | 3.4 | 107.2 | 4.2 |
|  | -6 | 114.5 | 0.4 | 108.5 | 0.9 |
|  | -7 | 108.1 | 3.4 | 109 | 1 |
|  | -8 | 101.7 | 3 | 106 | 1.7 |

**Table S4A. Systemic/organ specific toxicity parameters obtained in a combined repeated dose and reproduction/developmental toxicity screening test (OECD TG 422).**

^§^p<=0.05 (Dunnett test (two-sided) vs. control group.

^*^p<=0.05, ^**^p<=0.01 (Kruskal-Wallis test & Wilcoxon test; two-sided for clinical chemistry parameters) vs. control group.

|  |  |  | **F0 Males** | | | | | | | | **F0 Females** | | | | | | | |
| --- | --- | --- | --- | --- | --- | --- | --- | --- | --- | --- | --- | --- | --- | --- | --- | --- | --- | --- |
| **Endpoint** | | | **Negative vehicle control** | | **Linalool [mg/kg/d]** | | | | | | **Negative vehicle control** | | **Linalool [mg/kg/d]** | | | | | |
|  |  |  |  |  | **50** | | **200** | | **800** | |  |  | **50** | | **200** | | **800** | |
|  |  |  | mean | +/- SD | mean | +/- SD | mean | +/- SD | mean | +/- SD | mean | +/- SD | mean | +/- SD | mean | +/- SD | mean | +/- SD |
| Clincial signs |  |  | - |  | - | - | Salivation | - | Salivation, Unstready gait, Piloerection | - | - | - | - | - | Salivation | - | Salivation, Hyperexcitability, Unstready gait, Abdominal position, Piloerection, Pale skin, Semi-/closed eyelids, Smeared fur | - |
| Food consumption | Premating d0->13 | [g] | 19.1 | 1.3 | 20.0 | 2.3 | 19.8 | 0.9 | **16.1^§^** | 0.9 | 12.9 | 1.1 | 12.5 | 0.3 | 13.0 | 0.7 | 11.7 | 0.8 |
|  | Gestation d0->20 | [g] | - | - | - | - | - | - | - | - | 17.9 | 1.4 | 18.0 | 1.8 | 18.3 | 0.7 | 17.6 | 1.1 |
|  | Lactation d0->21 | [g] | - | - | - | - | - | - | - | - | 44.9 | 1.9 | 44.3 | 3.1 | 44.2 | 3.2 | **38.4^§^** | 8.0 |
| Body weights | Day 7 | [g] | 415.8 | 16.6 | 415.6 | 23.6 | 411.6 | 21.6 | 396.8 | 19.6 | - | - | - | - | - | - | - | - |
|  | Day 13 | [g] | 422.9 | 16.5 | 426.5 | 25.7 | 417.8 | 22.4 | 400.0 | 19.3 | - | - | - | - | - | - | - | - |
|  | Day 21 | [g] | 431.9 | 15.7 | 432.2 | 28.3 | 425.6 | 24.5 | **405.2^§^** | 19.8 | - | - | - | - | - | - | - | - |
|  | Premating Day 13 | [g] | - | - | - | - | - | - | - | - | 223.0 | 9.2 | 221.0 | 9.1 | 219.3 | 7.3 | 221.2 | 11.7 |
|  | Gestation Day 20 | [g] | - | - | - | - | - | - | - | - | 339.3 | 16.1 | 331.5 | 24.1 | 336.7 | 7.7 | 329.5 | 12.7 |
|  | Lactation Day 1 | [g] | - | - | - | - | - | - | - | - | 266.8 | 11.0 | 262.6 | 16.0 | 262.2 | 9.1 | **251.8^§^** | 10.4 |
|  | Lactation Day 4 | [g] | - | - | - | - | - | - | - | - | 280.7 | 12.4 | 274.3 | 14.4 | 280.0 | 8.8 | **266.1^§^** | 15.6 |
|  | Lactation Day 13 | [g] | - | - | - | - | - | - | - | - | 290.5 | 12.6 | 283.4 | 12.6 | 289.5 | 6.3 | 279.8 | 17.6 |
|  | Lactation Day 21 | [g] | - | - | - | - | - | - | - | - | 272.3 | 10.0 | 271.8 | 12.5 | 272.5 | 10.0 | 273.4 | 15.8 |
| Clinical chemistry | Calcium | [mmol/L] | 2.60 | 0.03 | 2.60 | 0.02 | 2.56 | 0.04 | **2.67*** | 0.06 | 2.34 | 0.05 | 2.40 | 0.08 | **2.45*** | 0.06 | **2.48*** | 0.11 |
|  | Sodium | [mmol/L] | 144.0 | 0.9 | 143.5 | 0.8 | 144.6 | 1.2 | 143.3 | 0.5 | 139.8 | 1.3 | 140.6 | 1.9 | 138.2 | 1.3 | **142.4*** | 1.4 |
|  | Urea | [mmol/L] | 4.01 | 0.49 | 4.49* | 0.15 | 4.27 | 0.81 | **4.92*** | 0.50 | 9.7 | 0.8 | 10.2 | 1.2 | 10.0 | 0.8 | 10.0 | 0.7 |
|  | Total protein | [g/L] | 63.11 | 2.03 | 63.45 | 2.58 | 62.93 | 2.51 | **71.00**** | 3.47 | 55.3 | 1.9 | 56.5 | 1.5 | 56.9 | 2.6 | 58.5 | 2.7 |
|  | Albumin | [g/L] | 39.35 | 1.35 | 39.41 | 1.17 | 39.31 | 1.02 | **44.77**** | 1.26 | 36.7 | 0.9 | 36.4 | 1.1 | 36.8 | 1.5 | 37.6 | 1.9 |
|  | Cholesterol | [mmol/L] | 1.74 | 0.15 | 1.91 | 0.36 | 1.72 | 0.23 | **1.25**** | 0.13 | 2.06 | 0.19 | 2.04 | 0.28 | 1.94 | 0.24 | 2.19 | 0.50 |
| Organ weight | Kidney(F0) | absolute (g) | 2.586 | 0.253 | 2.602 | 0.264 | 2.578 | 0.287 | 2.800 | 0.101 | 1.658 | 0.167 | 1.658 | 0.092 | 1.684 | 0.153 | 1.796 | 0.133 |
|  |  | relative (%) | 0.605 | 0.050 | 0.611 | 0.047 | 0.615 | 0.042 | **0.726**** | 0.043 | 0.693 | 0.063 | 0.690 | 0.031 | 0.709 | 0.074 | 0.755 | 0.045 |
| Histology | Kidney(F0) | Examined [n] | 10 | - | 10 | - | 10 | - | 10 | - | 5 | - | 1 | - | 0 | - | 5 | - |
|  |  | Casts; (multi)focal | 2 | - | 1 | - | 1 | - | 2 | - | 3 | - | 0 | - | - | - | 0 | - |
|  |  | Droplets, eosinophilic | 10 | - | 10 | - | 10 | - | 10 | - | 0 | - | 0 | - | - | - | 0 | - |
|  |  | Tubules, basophilic; (multi)focal | 2 | - | 7 | - | 8 | - | 8 | - | 2 | - | 0 | - | - | - | 2 | - |

**Table S4B. Reproductive toxicity parameters obtained in a combined repeated dose and reproduction/developmental toxicity screening test (OECD TG 422).**

^§^p<=0.05 (Fisher's exact test; one-sided+) versus control group based on number of litters with stillborn pups.

^$^p<=0.05 (Wilcoxon with Bonferroni-Holm; one-sided-) versus control group.

^**^p<=0.01 (Dunnett test (two-sided) vs. control group.

| **Endpoint** | | | **Negative vehicle control** | | **Linalool [mg/kg/d]** | | | | | |
| --- | --- | --- | --- | --- | --- | --- | --- | --- | --- | --- |
|  |  |  |  |  | **50** | | **200** | | **800** | |
|  |  |  | mean | +/- SD | mean | +/- SD | mean | +/- SD | mean | +/- SD |
| Female/male mating index | | % | 100 | - | 100 | - | 100 | - | 100 | - |
| Female/male fertility index | | % | 100 | - | 100 | - | 90 | - | 100 | - |
| Mating period | | days | 2.7 | 1.4 | 2.1 | 1.3 | 4.1 | 5.3 | 2.4 | 1.4 |
| Gestation period | | days | 22.3 | 0.5 | 22.1 | 0.3 | 22.0 | 0.0 | 22.6 | 0.7 |
| Implantation sites | | per dam | 13.1 | 1.1 | 12.0 | 4.4 | 12.3 | 3.2 | 13.5 | 1.6 |
| Postimplantation loss | | % | 8.3 | 7.2 | 16.2 | 30.0 | 5.0 | 7.6 | 14.1 | 15.8 |
| Pregnant but not delivering | | dam | 0 | - | 1 | - | 0 | - | 0 | - |
| Pups delivered | | per dam | 12.0 | 1.2 | 12.3 | 2.3 | 11.7 | 3.1 | 11.5 | 2.1 |
| Gestation index | | % | 100 | - | 90 | - | 100 | - | 90 | - |
| Live birth index | pups liveborn | % | 100 | - | 99.1 | - | 100 | - | **85.2** | - |
| Pups stillborn | | % | 0 | - | 0.9 | - | 0 | - | **14.8** | - |
| Litters | with stillborn pups | % | 0 | - | 11.1 | - | 0 | - | **40^§^** | - |
|  | with all pups stillborn | % | 0 | - | 0 | - | 0 | - | **10** | - |
| Viability index | PND 0-4 | % | 100 | 0 | 100 | 0 | 100 | 0 | **82.7^$^** | 24.2 |
| Lactation index | PND 4-21 | % | 74.6 | 5.0 | 74.6 | 1.2 | 74.1 | 2.8 | 72.2 | 8.3 |
| Pup body weights | day 21 (males + females) | g | 57.7 | 2.7 | 56.5 | 1.5 | 56.8 | 2.7 | **49.1**** | 4.4 |
| Pup body weight change | day 1-21 (males + females) | g | 50.7 | 2.3 | 49.8 | 1.5 | 50.0 | 2.3 | **43.1**** | 3.7 |

**Table S4C. Thyroid related parameters (incl. liver weights) obtained in a combined repeated dose and reproduction/developmental toxicity screening test (OECD TG 422).**

^**^p<=0.01 (Kruskal-Wallis test & Wilcoxon test; two-sided for hormone measurements) vs. control group; n.d.= not determined.

|  |  |  | **Males** | | | | | | | | **Females** | | | | | | | |
| --- | --- | --- | --- | --- | --- | --- | --- | --- | --- | --- | --- | --- | --- | --- | --- | --- | --- | --- |
| **Endpoint** | | | **Negative vehicle control** | | **Linalool [mg/kg/d]** | | | | | | **Negative vehicle control** | | **Linalool [mg/kg/d]** | | | | | |
|  |  |  |  |  | **50** | | **200** | | **800** | |  |  | **50** | | **200** | | **800** | |
|  |  |  | mean | +/- SD | mean | +/- SD | mean | +/- SD | mean | +/- SD | mean | +/- SD | mean | +/- SD | mean | +/- SD | mean | +/- SD |
| **Hormone - T4** | F0 | nmol/L | 60.89 | 5.74 | 60.23 | 7.06 | 62.59 | 7.06 | **36.39**** | 10.78 | n.d. | - | n.d. | - | n.d. | - | n.d. | - |
|  | F1 (PND13) | nmol/L | 58.29 | 9.51 | 55.64 | 10.65 | 56.97 | 9.02 | 54.00 | 15.99 | 59.32 | 11.58 | 53.26 | 11.96 | 56.88 | 8.57 | 48.17 | 12.84 |
| **Hormone - TSH** | F0 | µg/L | 7.08 | 3.81 | 8.15 | 3.21 | 6.70 | 1.50 | 5.24 | 1.63 | n.d. | - | n.d. | - | n.d. | - | n.d. | - |
|  | F1 (PND13) | µg/L | 4.51 | 0.85 | 4.31 | 0.59 | 4.35 | 0.27 | 4.11 | 1.13 | 4.58 | 1.55 | 4.13 | 0.89 | 4.32 | 0.72 | 4.55 | 1.02 |
| **Organ weight** | Liver (F0) | absolute (g) | 9.488 | 0.462 | 9.784 | 1.397 | 9.614 | 1.109 | 11.366 | 0.778 | 5.86 | 0.349 | 5.862 | 0.148 | 6.026 | 0.256 | **7.202**** | 0.526 |
|  |  | relative (%) | 2.222 | 0.055 | 2.288 | 0.122 | 2.293 | 0.143 | **2.941**** | 0.066 | 2.449 | 0.088 | 2.445 | 0.152 | 2.535 | 0.106 | **3.029**** | 0.174 |
|  | Thyroid (F0) | absolute (mg) | 24.9 | 4.4 | 25.1 | 4.1 | 26.0 | 4.9 | 21.0 | 4.8 | 17.2 | 3.6 | 17.4 | 1.4 | 18.4 | 3.2 | 17.3 | 2.4 |
|  |  | relative (%) | 0.006 | 0.001 | 0.006 | 0.001 | 0.006 | 0.001 | 0.006 | 0.001 | 0.007 | 0.002 | 0.007 | 0.001 | 0.008 | 0.001 | 0.007 | 0.001 |
| **Histology** | Thyroid (F0) | Examined [n] | 10 | - | 10 | - | 10 | - | 10 | - | 10 | - | 10 | - | 10 | - | 10 | - |
|  |  | Hypertrophy/ hyperplasia, follicular cell | 2 | - | 0 | - | 2 | - | 2 | - | 3 | - | 2 | - | 0 | - | 2 | - |
|  |  | Alteration, colloid | 1 | - | 0 | - | 0 | - | 1 | - | 0 | - | 0 | - | 0 | - | 0 | - |
